# Compartment-driven imprinting of intestinal CD4 T cells in inflammatory bowel disease and homeostasis

**DOI:** 10.1093/cei/uxad095

**Published:** 2023-08-11

**Authors:** Lisanne Lutter, José J M ter Linde, Eelco C Brand, David P Hoytema van Konijnenburg, Britt Roosenboom, Carmen Horjus Talabur-Horje, Bas Oldenburg, Femke van Wijk

**Affiliations:** Centre for Translational Immunology, University Medical Centre Utrecht, Utrecht, The Netherlands; Department of Gastroenterology and Hepatology, University Medical Centre Utrecht, Utrecht, The Netherlands; Centre for Translational Immunology, University Medical Centre Utrecht, Utrecht, The Netherlands; Department of Gastroenterology and Hepatology, University Medical Centre Utrecht, Utrecht, The Netherlands; Centre for Translational Immunology, University Medical Centre Utrecht, Utrecht, The Netherlands; Department of Gastroenterology and Hepatology, University Medical Centre Utrecht, Utrecht, The Netherlands; Centre for Translational Immunology, University Medical Centre Utrecht, Utrecht, The Netherlands; Division of Immunology, Boston Children’s Hospital and Department of Pediatrics, Harvard Medical School, Boston, MA, USA; Department of Gastroenterology and Hepatology, Rijnstate Hospital, Arnhem, The Netherlands; Department of Gastroenterology and Hepatology, Rijnstate Hospital, Arnhem, The Netherlands; Department of Gastroenterology and Hepatology, University Medical Centre Utrecht, Utrecht, The Netherlands; Centre for Translational Immunology, University Medical Centre Utrecht, Utrecht, The Netherlands

**Keywords:** inflammatory bowel diseases, small intestine, CD4 T cells, adaptation, RNA-sequencing

## Abstract

The mucosal immune system is implicated in the etiology and progression of inflammatory bowel diseases. The lamina propria and epithelium of the gut mucosa constitute two separate compartments, containing distinct T-cell populations. Human CD4 T-cell programming and regulation of lamina propria and epithelium CD4 T cells, especially during inflammation, remain incompletely understood. We performed flow cytometry, bulk, and single-cell RNA-sequencing to profile ileal lamina propria and intraepithelial CD4 T cells (CD4CD8αα, regulatory T cells (Tregs), CD69^−^ and CD69^high^ Trm T cells) in controls and Crohn’s disease (CD) patients (paired non-inflamed and inflamed). Inflammation results in alterations of the CD4 T-cell population with a pronounced increase in Tregs and migrating/infiltrating cells. On a transcriptional level, inflammation within the epithelium induced T-cell activation, increased IFNγ responses, and an effector Treg profile. Conversely, few transcriptional changes within the lamina propria were observed. Key regulators including the chromatin remodelers *ARID4B* and *SATB1* were found to drive compartment-specific transcriptional programming of CD4 T(reg) cells. In summary, inflammation in CD patients primarily induces changes within the epithelium and not the lamina propria. Additionally, there is compartment-specific CD4 T-cell imprinting, driven by shared regulators, between the lamina propria and the epithelium. The main consequence of intraepithelial adaptation, irrespective of inflammation, seems to be an overall dampening of broad (pro-inflammatory) responses and tight regulation of lifespan. These data suggest differential regulation of the lamina propria and epithelium, with a specific regulatory role in the inflamed epithelium.

## Teaser

Inflammation within the epithelium induced CD4 T-cell activation, increased IFNγ responses, and an effector Treg profile, whereas few transcriptional changes within the lamina propria were observed. Key regulators including the chromatin remodelers ARID4B and SATB1 were found to drive compartment-specific transcriptional programming of CD4 T(reg) cells. The main consequence of intraepithelial adaptation, irrespective of inflammation, seems to be an overall dampening of broad (pro-inflammatory) responses and tight regulation of lifespan.

## Introduction

Inflammatory bowel disease (IBD), comprising Crohn’s disease (CD) and ulcerative colitis, is a chronic relapsing-remitting inflammatory disease with a multifactorial and not fully elucidated pathogenesis [1]. Both the innate and adaptive immune systems have been implicated in the etiology and progression of the disease [1]. The lamina propria and epithelium of the gut mucosa constitute two separate compartments, containing distinct populations of T cells [2]. Intraepithelial T cells, interspersed between the single layer of epithelial cells, are in direct contact with food and microbiota-derived antigens, whereas T cells in the lamina propria are surrounded by a variety of other immune and stromal cells. In the lamina propria, most T cells are of the CD4 lineage, while CD8 T cells predominate in the epithelium [3]. CD4 T cells are commonly divided into T helper (Th, effector) cells, regulatory T cells (Tregs), and CD4CD8αα T cells, with many of these being considered residents of the mucosal environment (tissue-resident memory T (Trm) cells, expressing CD69) [3, 4]. In CD patients, CD4 T cells are increased in both the lamina propria and epithelium [5–8] and changes in the phenotypic profile of several CD4 T cell subsets have been observed, including an increased percentage of (double/multiple) cytokine-producing CD4 T cells (both regulatory and pro-inflammatory) and a more cytotoxic profile of these CD4 T cells [9–14]. Additionally, it is now clear that microenvironmental cues can steer functional programs and cell dynamics that might have a role in health and disease. However, since studies reporting on CD4 T cells primarily focused on lamina propria or on the bulk of mucosal cells, our understanding of CD4 T-cell programming in different mucosal compartments remains incompletely understood. This includes the programming of Tregs and CD4CD8αα T cells, both associated with regulation [15, 16], and the differential regulation of CD4 T cells derived from the lamina propria and epithelium, especially in inflammation. Studying tissue- and compartment-specific resident CD4 T-cell regulation in IBD may improve our understanding of chronic and locally recurrent inflammation. Moreover, focusing on the shared concepts in a heterogeneous disease such as IBD could open up new avenues for targeted treatment.

To assess these questions, we profiled surface marker-based CD4 T-cell subsets with bulk RNA-sequencing, complemented with an unbiased CD4 T-cell single-cell RNA-sequencing exploration, to map the CD4 T-cell compartment in the epithelium and lamina propria of the human ileum in control subjects and paired inflamed and non-inflamed ileal biopsies from patients with CD. Our results uncover compartment-specific CD4 T-cell imprinting upon translocation from the lamina propria to the epithelium driven by key regulators regulating effector function and lifespan in the epithelium.

## Materials and methods

### Participant inclusion

Patients with Crohn’s disease were prospectively enrolled at the Department of Gastroenterology and Hepatology, University Medical Center Utrecht and the outpatient clinic of the Rijnstate Crohn and Colitis Centre, Arnhem, The Netherlands. During ileocolonoscopy multiple biopsy specimens were taken for bulk (*n* = 4) and single-cell (*n* = 4) RNA-sequencing of sorted subsets. Non-inflamed biopsies were taken>2 cm of macroscopically visible inflamed ileum. Control subjects for bulk RNA-sequencing (*n* = 3) did not have a diagnosis of IBD and underwent ileocolonoscopy for polyp surveillance and had normal macroscopical ileal mucosa (see Table 1 for participant characteristics). Sex was not taken into account when selecting patients due to the sample size which would not allow for correction of the influence of sex on the immune system.

**Table 1.** Baseline patient characteristics

	Flow cytometric analysis		Bulk RNA-seq		scRNA-seq
	CD patients (*n* = 8)	C subjects (*n* = 5)	CD patients (*n* = 4)	C subjects (*n* = 3)	CD patients (*n* = 4)
Gender					
Female	2 (25)	4 (80)	1 (25)	3 (100)	4 (100)
Male	6 (75)	1 (20)	3 (7)	0 (0)	0 (0)
Age, years	39 (29-49)	57 (53-60)	36 (29-43)	56 (55-58)	44 (30-56)
Treatment at ileocolonoscopy					
None	3 (37.5)	5 (100)	2 (50)	3 (100)	3 (75)
5-ASA	0 (0)	0 (0)	0 (0)	0 (0)	1 (2)
Steroids	1 (12.5)	0 (0)	0 (0)	0 (0)	0 (0)
Thiopurine	2 (25)	0 (0)	1 (25)	0 (0)	0 (0)
Thiopurine + anti-TNF	1 (12.5)	0 (0)	0 (0)	0 (0)	0 (0)
Anti-TNF	1 (12.5)	0 (0)	1 (25)	0 (0)	0 (0)
Anti-IL12/23	0 (0)	0 (0)	0 (0)	0 (0)	0 (0)
SES-CD score				NA	
Inactive disease	0 (0)		0 (0)		0 (0)
3–6 Mild disease	0 (0)		0 (0)		1 (25)
7–15 Moderate disease	2 (25)		0 (0)		0 (0)
≥16 Severe disease	0 (0)		0 (0)		1 (25)
Rutgeerts score		NA		NA	
i0	1 (12.5)		0 (0)		0 (0)
i1	0 (0)		0 (0)		0 (0)
i2	4 (50)		3 (75)		2 (50)
i3	0 (0)		0 (0)		0 (0)
i4	1 (12.5)		1 (25)		0 (0)
Montreal CD		NA		NA	
Location					
L1: ileal	4 (50)		2 (50)		1 (25)
L2: colonic	0 (0)		1 (25)		1 (25)
L3: ileocolonic	4 (50)		1 (25)		2 (50)
Behavior					
B1: nonstricturing, nonpenetrating	5 (62.5)		2 (50)		1 (25)
B2: structuring	2 (25)		1 (25)		3 (75)
B3: penetrating	1 (12.5)		1 (25)		0 (0)
Perianal	4 (50)		2 (50)		0 (0)

Values expressed in *n* (%) or as median with interquartile range. The SES-CD score or the Rutgeerts score was assessed, depending on whether it concerned a post-surgical assessment (Rutgeerts score) or not (SES-CD); hence, the total percentage per scoring system does not necessarily add up to 100%. CD: Crohn’s disease; C: control; RNA-seq: RNA-sequencing; sc: single cell; SES-CD: simple endoscopic score for CD.

### Enzymatic digestion

Biopsies were collected in HBSS media (Gibco) containing 2% FCS and 0.2% amphotericin B. The intestinal tissue was transferred to HBSS supplemented with 1 mM DTT (Sigma) and placed on a rolling device for 10 min at 4°C. After discarding the supernatant, the intestinal tissue was transferred to HBSS supplemented with 2% FCS and 5 mM EDTA and shaken (2×) at 180 rpm for 30 min at 37°C. The tissue suspension was passed through a 70 µm cell strainer (Costar) and constituted the intraepithelial population. To obtain lamina propria T cells, the remaining intestinal biopsies were subsequently incubated for 1 h at 37°C with 1 mg/ml collagenase IV (Sigma) in RPMI medium (supplemented with 10% FCS, 100 U/ml penicillin–streptomycin, and 0.2% Fungizone), then forcefully resuspended through a 19G needle, washed and filtered with 70 µm cell strainer (Costar). The cell suspensions were used for bulk and single-cell RNA-sequencing after sorting different T-cell subsets.

### Fluorescence-activated cell sorting

In preparation for fluorescence-activated cell sorting (FACS) the intestinal cells were incubated with the surface antibodies for 20 min in supplemented RPMI (2% FCS, 1% penicillin and streptomycin, 0.2% Fungizone) at 4°C, and subsequently washed in FACS buffer before sorting on a FACSAria™ III (BD) in TRIzol LS (Thermo Fisher Scientific) and immediately frozen at −80°C (for gating strategy, see Supplementary Fig. S1A). Antibodies used: fixable viability dye eF506 (65-2860-40), anti-human CD3 eF450 (clone OKT3, 48-0037-42; eBioscience), TCRγδ BV510 (clone B1, 331220), CD3 AF700 (clone UCHT1, 300424), CD4 BV785 (clone OKT4, 317442; Biolegend), CD8α APC-Cy7 (clone SK1, 557834), CD127 BV421 (clone HIL-7R-M21, 562436), CD25 PE-Cy7 (clone M-A251, 557741), CD69 PE (clone FN50, 555531; BD Biosciences), CD8α PerCP-Cy5.5 (clone RPA-T8, 301032), CD127 AF647 (clone HCD127, 351318; Biolegend), TCRγδ FITC (clone IMMU510, IM1571U, Beckman Coulter), CD45RA APC-Cy7 (clone HI100, 2120640), and CCR7 APC (clone G043H7, 2366070, Sony Biotechnology). Flow data was analyzed using FlowJo v10.

### Bulk RNA-sequencing

The sorted cells were thawed in TRIzol LS for TRIzol (Thermo Fisher Scientific) RNA extraction and stored at −80°C until library preparation. Sequencing libraries were prepared using the Cel-Seq2 sample preparation protocol and sequenced as 75 bp paired-end on a NextSeq 500 (Utrecht sequencing facility). The reads were demultiplexed and aligned to the human cDNA reference genome (hg38) using BWA (version 0.7.13). Multiple reads mapping to the same gene with the same unique molecular identifier (UMI, 6 bp long) were counted as a single read.

Raw counts of splice variants were summed, and the raw counts were subsequently transformed employing variance stabilizing transformation. Differential analysis was performed using DESeq2 (Wald’s test) with a *P*-adjusted value <0.1 considered statistically significant. For visualization purposes the R package DESeq2 was employed. Pathway analysis was performed on the differentially expressed genes as input in Toppfun with standard settings. Gene set enrichment analysis (GSEA v4.0.3 [17]), with as input the normalized data (output DESeq2), was used to assess the enrichment of gene sets derived from Magnuson *et al*. [18], Guo *et al*. [19], and Mijnheer *et al*. [20]. One thousand random permutations of the gene sets were used to establish a null distribution of enrichment scores against which a normalized enrichment score and FDR-corrected *q* values were calculated. Identification of key-regulators was performed using RegEnrich v1.0.1 [21] based on the differential gene expression data followed by unsupervised gene regulatory network inference (GENIE3) to construct a network based on the raw gene count data, and GSEA was used for enrichment analysis. Network visualization was performed with Cytoscape v3.9.0 [22].

### Single-cell RNA-sequencing

Live CD3^+^CD8^-^CD4^+^ cells were sorted (for gating strategy see Supplementary Fig. S1A) into 384-well hard shell plates (Biorad) with 5 μl of vapor-lock (QIAGEN) containing 100–200 nl of RT primers, dNTPs and synthetic mRNA Spike-Ins and immediately spun down and frozen to −80°C. Cells were prepared for SORT-seq as previously described [23]. Illumina sequencing libraries were then prepared with the TruSeq small RNA primers (Illumina) and sequenced single-end at 75 bp read length with 75 000 reads per cell on a NextSeq500 platform (Illumina). Sequencing reads were mapped against the reference human genome (GRCh38) with BWA.

Quality control was performed in R with Seurat and cells were dropped when the number of genes was <150, and/or the percentage of mitochondrial genes was >35%. Potential doublets were eliminated based on the gene/UMI ratio. Cut-offs were set based on visual inspection of the distribution and preliminary clustering analyses. The raw data expression matrices were subsequently analyzed using Seurat (v4 [24–26]) following the outline provided by the distributor (https://satijalab.org/seurat/). Each dataset was log-normalized, variable features were determined using vst, and the percentage of mitochondrial genes and the difference between G1 and G2M phases of the cell-cycle were regressed out. Thereupon, the datasets were merged with Seurat and batch-effect correction for patients was performed with Harmony [27].

For dimensionality reduction, first the PCs were calculated (RunPCA) and clustering was performed with UMAP (RunUMAP: 30 dimensions, n.neighbors 20; FindNeighbors: clustering resolution of 0.8). Index sort data was analyzed in FlowJo v10 using the Index sort v3 script (https://www.flowjo.com/exchange/#/plugin/profile?id=20). Median fluorescent intensity cut-offs were determined based on the gating and imported as metadata. Subsequent differential gene expression was performed using the MAST test (standard settings, regression for patient) with a *P*-adjusted value <0.05 considered statistically significant. Visualization was performed with Seurat.

### Statistical analysis

Flow cytometric data was analyzed with a two-tailed Mann–Whitney *U* or Wilcoxon test with a *P*-adjusted value <0.05 considered statistically significant. Data were analyzed with GraphPad Prism (GraphPad Software version 7.0, La Jolla, CA, USA). Singe cell and bulk RNA-sequencing was statistically analyzed as described in the relevant paragraphs.

### Ethics and patient consent statements

The study protocols (TCBio 17/443, 17/444, and NL28761.091.09) were approved by the research ethics committee of the University Medical Center Utrecht (Utrecht, The Netherlands) and the Radboud University Nijmegen Medical Centre (CMO Regio Arnhem-Nijmegen, Nijmegen, The Netherlands), respectively. Written informed consent was obtained from each participating patient before any study-related procedure was performed. The procedures were performed in accordance with the Declaration of Helsinki.

## Results

### Increased epithelial and lamina propria regulatory and effector CD4 T cells in (active) ileal Crohn’s disease

**Figure 1.**
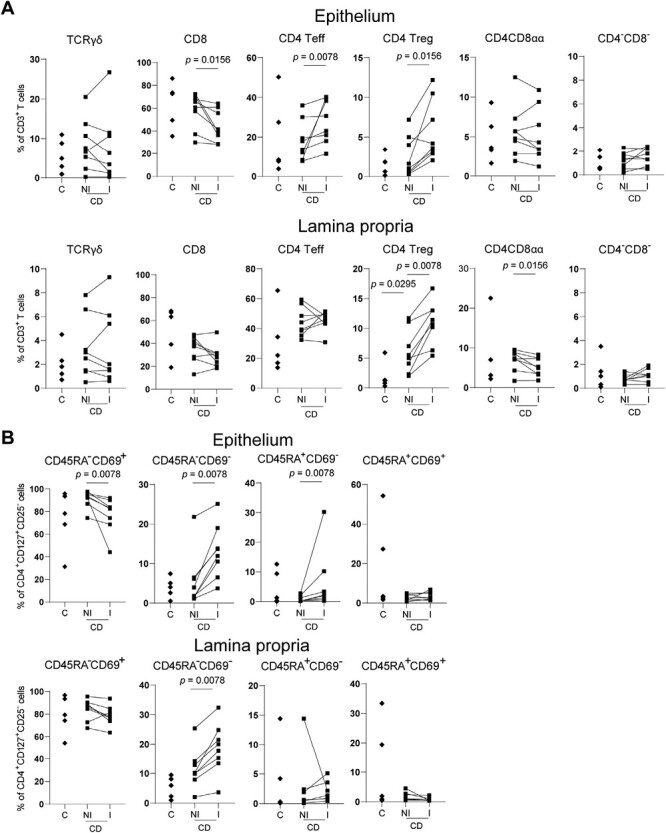
T-cell composition of the human ileum in health and Crohn’s disease. (**A**) Relative composition of T-cell receptor (TCR)γδ, CD8, CD4 effector (Teff, CD127^+^CD25^−^), CD4CD8αα (CD4^+^CD8α^+^), CD4 regulatory T cells (Tregs, CD127^low^CD25^high^), and TCRγδ^−^CD8^−^CD4^−^ cells in the epithelium (upper row) and lamina propria (lower row) of control subjects (C), non-inflamed (NI), and inflamed (I) ileum of Crohn’s disease (CD) patients. Lines connect the paired non-inflamed and inflamed datapoints from the patients with CD. Control subjects *n* = 5, CD *n* = 8. (**B**) Relative composition within CD4 effector T cells of tissue-resident memory (CD45RA^−^CD69^+^), memory (CD45RA^−^CD69^−^), CD4 naive (CD45RA^+^CD69^−^), and recently activated (CD45RA^+^CD69^+^) T cells, similar to A. Control subjects *n* = 5, CD *n* = 8. Comparisons were performed with a two-tailed Mann–Whitney *U* test for control subject versus non-inflamed CD and Wilcoxon test for paired non-inflamed versus inflamed CD

We first analyzed the composition of major T-cell populations in the epithelium and lamina propria of the human ileum. Therefore, we obtained ileal biopsies from control subjects as well as paired non-inflamed and inflamed ileal biopsies from patients with CD (gating strategy: Supplementary Fig. S1A). We assessed the presence of the following T-cell subsets: T-cell receptor (TCR)γδ, TCRγδ^−^CD8^−^CD4^−^, CD8, CD4CD8α (of which the vast majority is expected to be CD4CD8αα), effector (CD4^+^CD127^+^CD25^−^), and Treg (CD4^+^CD127^low^CD25^high^) cells (Fig. 1A). The non-inflamed ileum of control subjects and patients with CD was found to display a comparable T-cell subset composition, except for an increase in Tregs in the lamina propria of patients with CD (*P *= 0.0295). Inflammation resulted in a further increase in Tregs (*P* = 0.0078) and a decrease in CD4CD8αα T cells (*P* = 0.0391) in the lamina propria compared to the paired non-inflamed ileum of patients with CD. The epithelium of the inflamed ileum of patients with CD displayed increased percentages of Tregs and CD4 effector T cells as well (*P* = 0.0156 and 0.0078, respectively), with a concomitant decrease in CD8 T cells (*P *= 0.0156) compared to control subjects. Furthermore, within the CD4 effector T-cell subset, an increase in CD69^−^ cells was observed in CD patients (*P *= 0.0451 for lamina propria CD45RA^−^CD69^−^ in CD versus control subjects) compared to control subjects, which was most pronounced in the inflamed epithelium (*P* = 0.0078 for CD45RA^−^CD69^−^ and CD45RA^+^CD69^−^ in inflamed versus non-inflamed epithelium and lamina propria, (Fig. 1B), indicating infiltration/expansion of CD69^low^ effector memory and naive CD4 T cells. Thus, most inflammation-associated changes in the overall T-cell composition impact the regulatory and migrating/infiltrating CD69^low^ CD4 effector subset content and occur within the epithelium.

### Inflammation is associated with an effector Treg profile in the epithelium.

**Figure 2.**
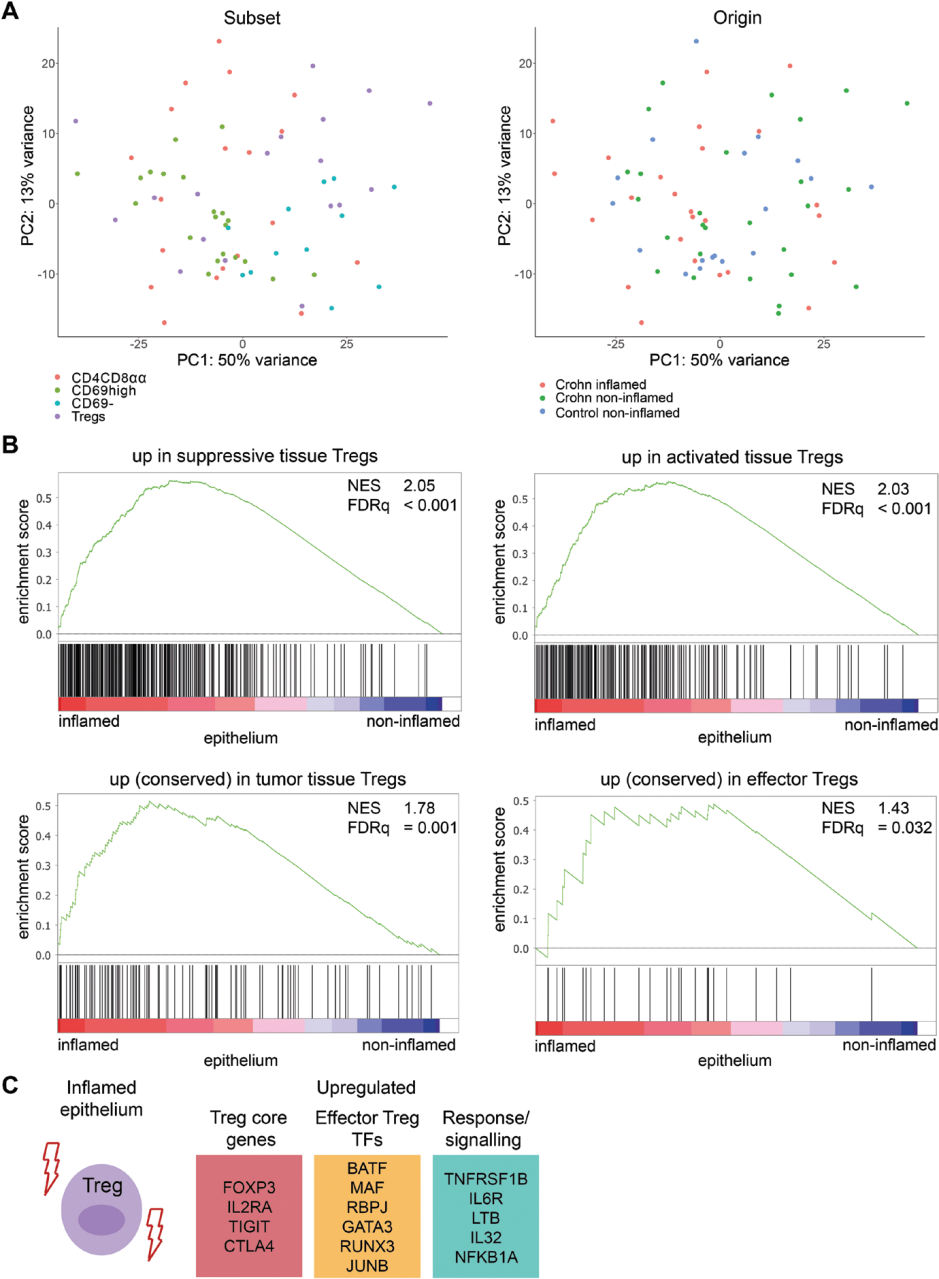
Effector Treg profile in the inflamed epithelium. (**A**) Unsupervised principal component analysis of all CD4 T-cell subsets analyzed by bulk RNA-sequencing colored on subset (ie, CD4CD8αα T cells, CD69^high^ Trm cells, CD69^−^ T cells and Tregs, left)), and status (ie, CD inflamed, CD non-inflamed, and  control non-inflamed; right). (**B**) Gene set enrichment analysis of suppressive [19] (upper left), activated [19] (upper right), tumor-derived tissue Treg [18] (lower left), and conserved human effector Treg [20] (lower right) signatures in pairwise comparisons of Tregs derived from non-inflamed and inflamed ileum of CD patients, represented by the normalized enrichment score (NES) and FDR statistical value (FDRq). (**C**) Selected genes upregulated in Tregs in the inflamed epithelium. TFs: transcription factors. CD inflamed/non-inflamed *n* = 4, control *n* = 3

We then performed bulk RNA-sequencing of sorted CD4CD8αα T cells, Tregs (CD4^+^CD127^low^CD25^+^), and effector T cells (CD4^+^CD127^+^CD25^-^) subdivided in CD69^−^ and CD69^high^ (Trm cells) from the ileum of three control subjects and four patients with CD (paired inflamed and non-inflamed; gating strategy: Supplementary Fig. S1A). Although principal component analysis revealed partial clustering of surface marker-based CD4 T-cell subsets, there was no complete distinction of all subsets (Fig. 2A, left; Supplementary Fig. 3A). For example, lamina propria CD4CD8αα T cells only differed from CD69^high^ Trm cells by expression of *CD8A*. In the epithelium, however, 13 upregulated and 138 downregulated genes were observed in CD4CD8αα T cells compared to CD69^high^ CD4 Trm cells, although none of these genes have clear effector, regulatory, nor cytotoxic, functions (Supplementary Table S1). In mouse epithelium, T-bet and Runx3 induce the intraepithelial CD4 T-cell program including CD8α expression [28]. Both T-bet and Runx3 were, however, not differentially expressed in human small intestinal CD4CD8αα T cells in the epithelium compared to the lamina propria, nor compared to CD69^high^ Trm cells or Tregs. CD4CD8αα T cells did differ from Tregs with downregulated *FOXP3*, and downregulation of the canonical Treg markers *IL2RA*, *IKZF2*, *TIGIT,* and *TNFRSF18* (Supplementary Table S1). This is in line with previous data [15, 29] and indicates that CD4CD8αα T cells are clearly different from mouse and human small intestinal FOXP3^+^ Tregs. As expected, the Trm signature was more enriched in CD69^high^ Trm compared to CD69^−^ CD4 T cells within the lamina propria (Supplementary Fig. S3B). Among the 395 upregulated and 31 downregulated genes were many known Trm genes (e.g. *CD69*, *CXCR6*, *IFNG,* and *ITGA1* [4]) (Supplementary Table S1).

Moreover, the origin of the samples (control subjects or patients with CD) was not found to fully drive clustering either, irrespective of the presence of inflammation (Fig. 2A, right; Supplementary Fig. S3A). First, we analyzed the impact of inflammation on the whole CD4 T-cell population (combined CD69^high^ Trm cells, CD4CD8αα T cells, and Tregs). Whereas only one differentially expressed gene was found when comparing CD4 T cells from paired inflamed and non-inflamed lamina propria of patients with CD, comparing CD4 T cells from inflamed lamina propria with healthy non-inflamed control subjects resulted in 1030 upregulated and 822 downregulated genes (Supplementary Table S1). Upregulated genes included *STAT1*, *BATF*, *PRDM1*, *GZMB*, *IL22,* and *CCR6*, and associated with processes related to cell activation, cell cycling, and stress. In the epithelium, significant differences in inflamed versus non-inflamed epithelium of patients with CD were found (1009 upregulated and 58 downregulated genes, Supplementary Table S1). Here, CD4 T cells from the inflamed epithelium showed, among others, increased translation and protein transport, T-cell activation, and responses to IFNγ, with upregulated genes including ribosomal genes, *CXCR3*, *CCR7*, *TOX2,* and *KLF2*.

Even though the relative composition within the CD4 T-cell population of the inflamed ileum shifted (Fig. 1A and B), only a limited number of transcriptional changes (<10) was found for any of the sorted bulk CD4 T-cell subsets (CD69^−^, CD69^high^, CD4CD8αα T cells, and Tregs) derived from inflamed ileum, except for intraepithelial Tregs. Intraepithelial Tregs from inflamed ileum mucosa acquired an effector Treg profile with upregulation of *FOXP3*, *TIGIT,* and *TNFRSF1B*, among others (Fig. 1B and C; Supplementary Table S1). Enrichment of gene signatures procured from literature for suppressive, activated, tumor suppressive Tregs and a conserved effector Treg program in inflammation-derived intraepithelial Tregs corroborate these observations (Fig. 2B) [18–20]. *FOXP3*, *GATA3*, *BATF*, *RUNX3,* and *MAF* were among the upregulated transcription factors (TFs) in Tregs from the inflamed epithelium which have been associated with effector Treg differentiation [20, 30–32]. Both GATA3 and RUNX3 are crucial in suppression of inflammation by Tregs [31, 32]. Thus, the most pronounced transcriptional change in CD4 T-cell subsets in CD inflammation is the strong effector Treg profile in the epithelial compartment.

### Mucosal sub-compartment drives the CD4 T-cell transcriptomic profile in the ileum

**Figure 3.**
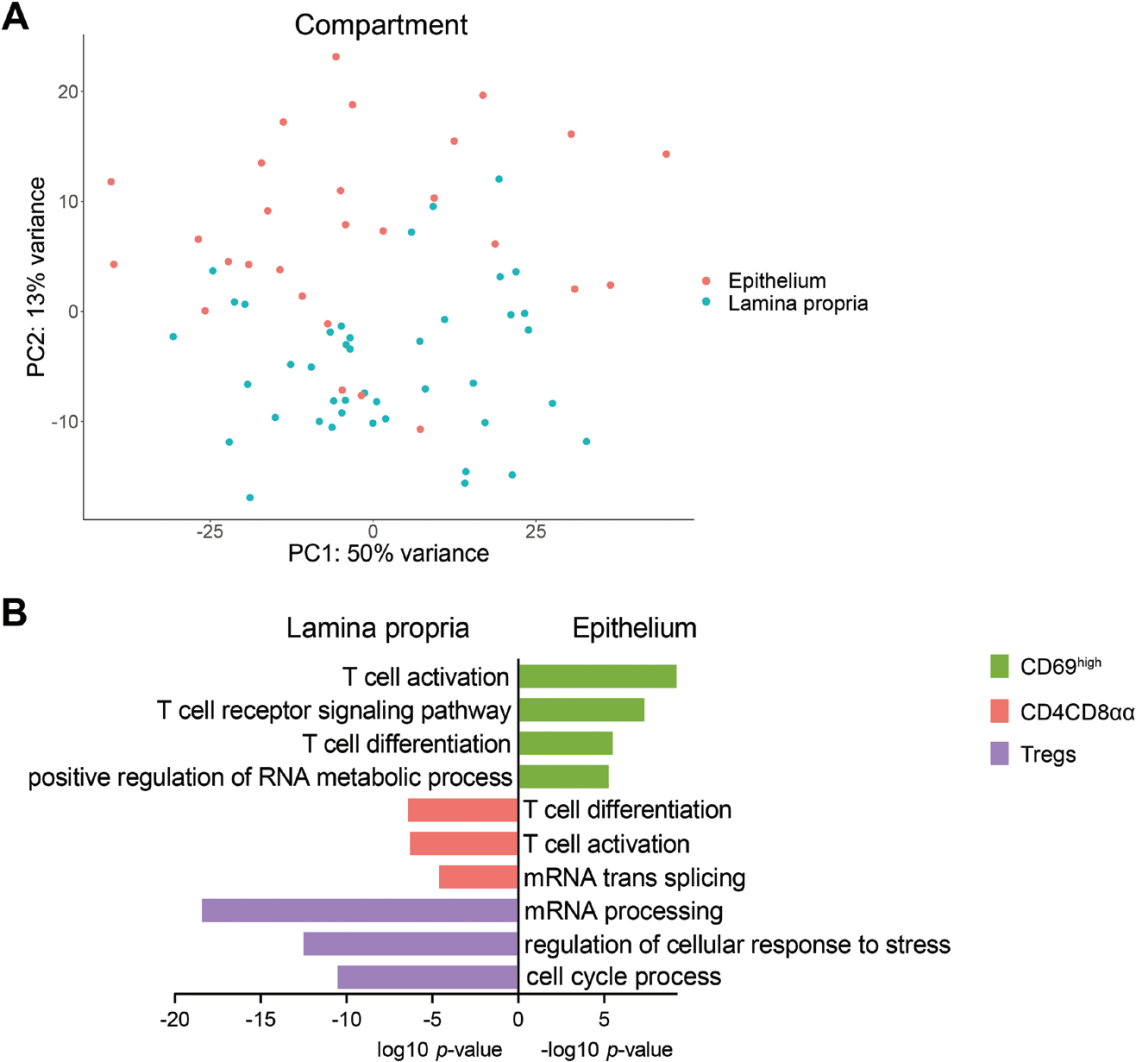
Mucosal sub-compartment shapes the transcriptomic profile of CD4 T cells. (**A**) Unsupervised principal component analysis of all CD4 T-cell subsets analyzed by bulk RNA-sequencing colored on compartment of residence (i.e. epithelium and lamina propria). (**B**) Selected biological process terms related to the upregulated genes in the lamina propria for CD69^high^ Trm cells, and in the epithelium for CD4CD8αα T cells and Tregs. CD inflamed/non-inflamed *n* = 4, control *n* = 3

Clear separation of CD4 T-cell subsets was observed based on the compartment of residence; the epithelium and lamina propria of the ileum (Fig. 3A, Supplementary Table S1). Gene ontology analysis revealed that CD69^high^ Trm cells in the epithelium were enriched for pathways associated with activation, T-cell differentiation, and mRNA processing (790 upregulated and 299 downregulated genes for epithelium versus lamina propria; Fig. 3B). However, pathways related to activation were upregulated in both lamina propria Tregs and CD4CD8αα T cells (708 and 239 upregulated and 1109 and 734 downregulated genes for epithelium versus lamina propria, respectively). These included cell activation and T-cell differentiation for CD4CD8αα T cells, and mRNA processing, stress response, and cell cycle regulation for Tregs (Fig. 3B). The enriched pathways for the different CD4 T-cell subsets were predominantly regulated via different genes (21 shared genes in the epithelium and 22 in the lamina propria). In the lamina propria, *TRAF1*, *TRAF4,* and *BIRC3* involved in TNF(R) signaling, as well as *IL4R* and *IL2RA* associated with T-cell activation and differentiation, were shared among all CD4 T-cell subsets [33], whereas in the epithelium genes involved in migration and motility were shared (e.g. *CLIC1* [34], *GNAQ* [35], *FAM107A* [36]) (Supplementary Table S1).

**Figure 4.**
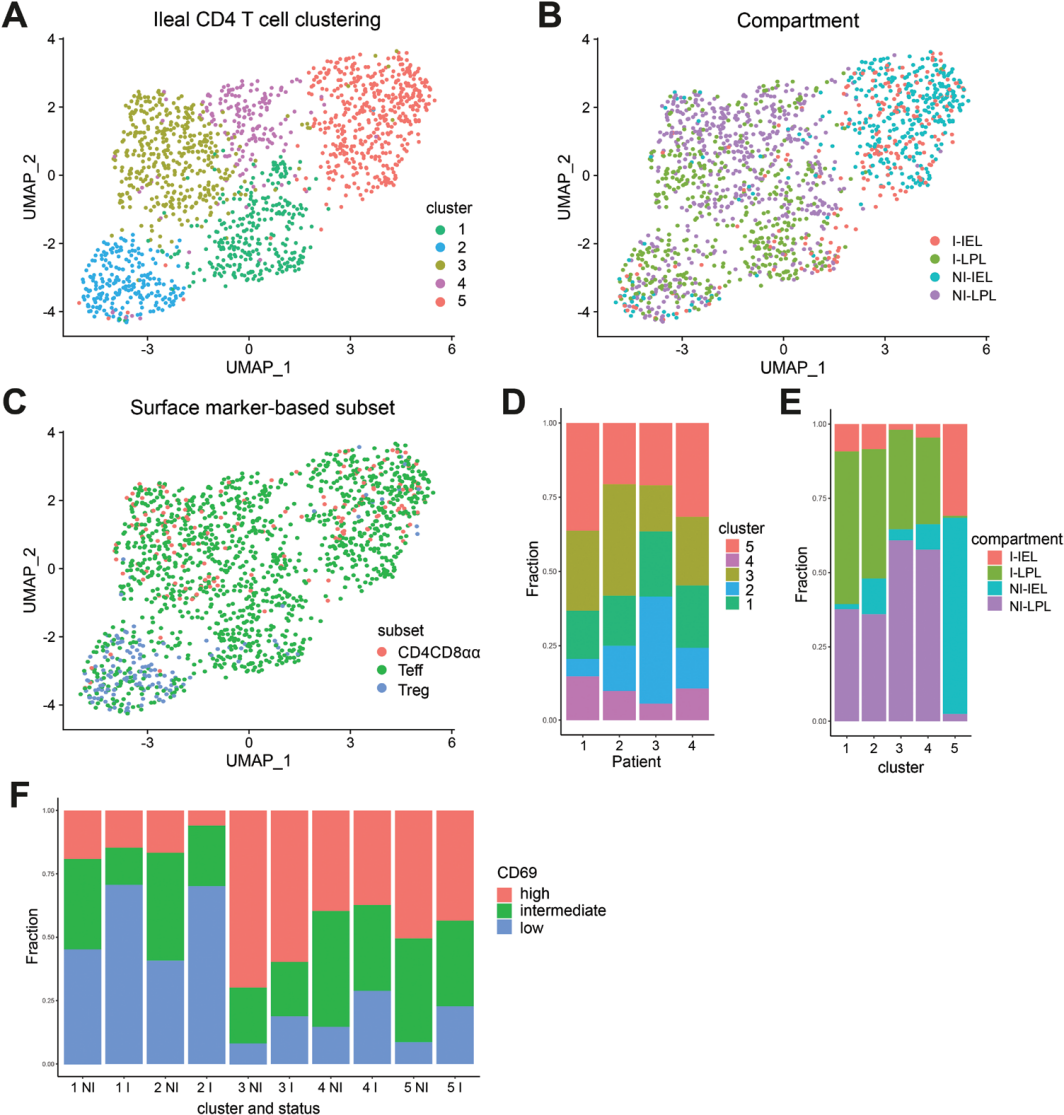
CD4 T-cell clusters in the paired non-inflamed and inflamed ileum. (**A**) Dimensionality reduction of all CD4 T cells (CD3^+^TCRγδ^−^CD4^+^) from the epithelium and lamina propria of four patients with Crohn’s disease. Cells are colored based on the assigned cluster. (**B**) As per A colored on the compartment (IEL = intraepithelial T cell, LPL = lamina propria T cell) and status (I = inflamed, NI = non-inflamed). (**C**) As per A colored on surface marker-based identification. CD4CD8αα T cell = CD4^+^CD8α^+^, effector T cell (Teff) = CD4^+^CD127^+^CD25^−^, Treg = CD127^−^CD25^+^. (**D**) Reproducible composition of the CD4 T cells across the four included patients. y-axis: fraction of cells colored on the cluster as shown in A and separated per patient on the x-axis. (**E**) Composition of the compartment (epithelium/lamina propria) and status (non-inflamed/inflamed) origins per cluster. y-axis: fraction of cells colored on compartment and separated per cluster as shown in A on the x-axis. (**F**) Composition of CD69 protein expression among non-inflamed (NI) and inflamed (I) derived CD4 T cells for all clusters, separated by low (negative), intermediate, and high CD69 expression. y-axis: fraction of cells colored on CD69 expression, and separated per cluster, and non-inflamed/inflamed ileum. CD inflamed/non-inflamed *n* = 4

**Figure 5.**
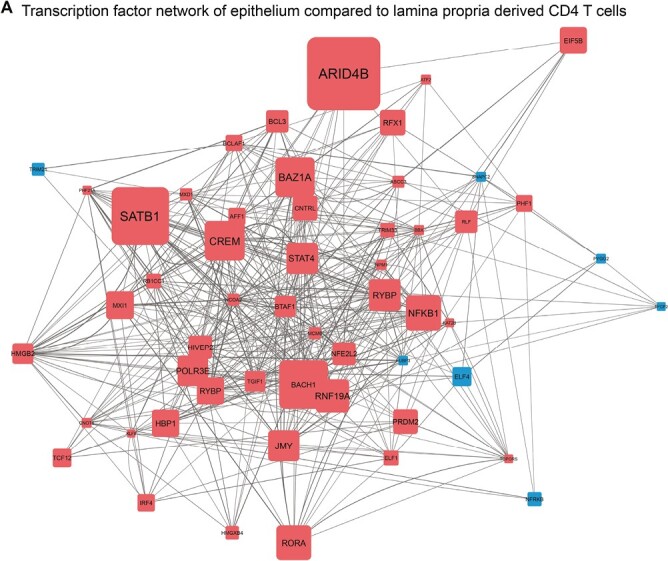
Predicted key regulators of epithelial adaptation of CD4 T cells. (**A**) Network inference of key-regulators driving adaptation of CD4 T cells to the epithelium on RNA level, based on unsupervised gene regulatory network analysis followed by gene set enrichment analysis of the transcription factors (TFs) and co-factors (pink = upregulation, blue = downregulation). The gray lines indicate connections between regulators (TFs) and their downstream targets (only TFs are shown); the line thickness represents the correlation weight (thicker = higher correlation). Square size indicates −log10(*p*) for each comparison, with the *P*(-value) derived from differential expression analysis (log2 fold change >0.5 and *P*-adjusted value <0.1); text size represents the RegEnrich score; for both, larger indicates higher scores (Supplementary Table S3). CD inflamed, non-inflamed, and control subjects combined *n* = 11

Subsequently, we performed single-cell RNA-sequencing of paired non-inflamed and inflamed ileum-derived CD4 T cells (CD3^+^TCRγδ^−^CD4^+^) from the epithelium and lamina propria of four patients with CD. Unsupervised clustering of 1573 cells after quality control supported our observation that the compartment is the primary driver of CD4 T-cell clustering (Fig. 4A–C, gating strategy: Supplementary Fig. S1A). Five CD4 T-cell clusters were detected (Fig. 4A and D). Recently migrated/recirculating T cells (cluster 1, 18.6% of all cells; *KLF2*, *SELL*, *CCR7*, *TCF7,* and *LEF1*) and Tregs (cluster 2, 14.3% of all cells; *FOXP3*, *TIGIT*, *CTLA4*, *IKZF2*, and *BATF*) comprised both lamina propria and intraepithelial cells (Fig. 4A and E). Furthermore, a mix of Th1- and Th17-skewed cells (cluster 3, 26.8% of all cells; *CCL20*, *NFKB1/2*, *TRAF1*, and *BHLHE40*) and cells expressing higher levels of heat shock protein family members (cluster 4, 11.1% of all cells) predominantly comprised lamina propria-derived CD4 T cells (Fig. 5A). The last cluster (cluster 5, 29.2% of all cells) comprised intraepithelial CD4 T cells distinguished by expression of innate and/or cytotoxic related genes including *FOS(B)*, *EGR1*, *GZMA*, *JUN*, and *IER2* (Fig. 5A and E, Supplementary Table S2). CD4CD8αα T cells mixed with the (resident) CD4 T cells and not with the Tregs, in line with the bulk RNA-sequencing data (Fig. 4C). Also, in accordance with the bulk data, inflammation did not result in distinct CD4 T-cell profiles, although, congruent with the protein data, we observed a relative increase in CD69^−/low^ CD4 T cells in all defined clusters in inflammation (Fig. 4F). Almost 50% of cluster 1 and 2 cells comprised CD69^low^ T cells, compared to ~10–15% for the other clusters, and these clusters showed the highest relative increase of CD69^low^ T cells in inflamed tissue. In summary, bulk and single-cell RNA-sequencing data revealed that the intra-tissue compartmentalization of CD4 T cells is the primary driver of their transcriptomic landscape. During inflammation, the expression profiles of CD4 Trm cells are largely preserved, with a significant influx of CD69^low^ migrating CD4 T cells.

### The chromatin remodeling genes ARID4B and SATB1 are predicted key regulators of lamina propria to epithelium translocation of CD4 T cells

Since we observed differences in the transcriptional profile of CD4 T cells derived from the lamina propria and epithelium we performed a data-driven network and enrichment analysis on the bulk RNA-sequencing data (RegEnrich [21]). Herewith, we could identify which key gene regulators, based on TFs and co-factors, differently drive lamina propria and epithelial compartment-specific adaptation (combined CD69^high^ Trm cells, CD4CD8αα T cells, and Tregs; *n* = 57), irrespective of inflammation. Top predicted regulators enabling adaptation of CD4 T cells to the epithelial compartment were *ARID4B*, *SATB1*, *CREM*, *BAZ1A,* and *NFKB1* (all negative, i.e. regulators upregulated in the lamina propria and downregulated in the epithelium; Fig. 5A, Supplementary Table 3). Most regulators were found to be downregulated. Of interest, downregulation of many of the abovementioned regulators is involved in chromatin remodeling and inhibits T-cell functioning. The negative regulator ARID4B is known as a master regulator of the phosphatidylinositol-3-kinase (PI3K) pathway related to T-cell activation and proliferation [37, 38]. Downstream target genes of the downregulated SATB1 are also involved in T-cell effector functions including *TRAF1* and *TRAF4* (TNF receptor-associated factors), *NFKB*, interleukin receptors (e.g. *IL4R*, *IL12RB2*), and *IL2*. Additionally, among the positive regulators were *ELF4*, *TRIM21,* and *TRIM27*, all linked to negative regulation of T-cell function. *ELF4* has been associated with CD8 T-cell quiescence and suppressed proliferation and downregulation of CCR7, CD62L, and KLF2 and thereby tissue retention [39]. *TRIM21* (Ro52) has been linked to negative regulation of pro-inflammatory cytokine production [40] by limiting Th1/Th17 differentiation and thereby suppressing tissue inflammation in an IL23/Th17-dependent manner [40, 41]. Lastly, *TRIM27* does not inhibit Th-cell differentiation but negatively impacts CD4 T-cell functioning via the PI3K pathway [42]. Furthermore, positively regulator-associated genes encompassed *FASLG* and programmed cell death genes, whereas negatively associated downstream genes included cell cycling genes. This suggests a different regulation of proliferation and cell death in these compartments. Altogether, the predicted network of TF regulators involved in the adaptation of CD4 T cells to the epithelium showed downregulation of chromatin remodeling, dampening of T-cell activation and effector function, as well as regulation of programmed/activation-induced cell death and arrest of cell-cycling independent of inflammation. Overall, these data suggest that function and lifespan are tightly regulated in intraepithelial CD4 T cells.

## Discussion

In the present study, we demonstrate that the transcriptional profile of CD4 effector, Trm cells, CD4CD8αα T cells, and Tregs is primarily determined by the compartment of residence (epithelium or lamina propria), irrespective of inflammation. The relative composition of the mucosal CD4 T-cell population changes in patients with CD with an increase in Tregs. Upon inflammation, Tregs as well as CD4 naive and migrating/infiltrating CD69^low^ T cells further increase, while the number of CD4 Trm cells, CD8, and CD4CD8αα T cells relatively decrease. These changes are most pronounced in the epithelium. Inflammation induces only few transcriptomic changes in lamina propria CD4 Trm cells, Tregs, and CD4CD8αα T cells on the bulk and single-cell RNA-sequencing level, indicating that reshaping of CD4 T-cell subset transcriptomes in the inflamed lamina propria of patients with CD is limited. In the inflamed epithelium, however, a considerable upregulation of cell activation, cell cycling, and stress response is observed, with Tregs gaining an effector Treg profile, which is also seen in tissues in other inflammatory diseases [20]. Adaptation of CD4 T cells to the epithelium, irrespective of inflammation, is driven by chromatin remodeling via *ARID4B* and *SATB1*, and the main consequence of intraepithelial adaptation seems to be a dampening of broad (pro-inflammatory) T-cell responses.

Previous studies have shown that T-cell subsets derived from the small intestine and colon are transcriptionally different, independent of inflammation [43]. Additionally, research has indicated that there are less differences between T-cell subsets (CD4 and CD8 effector T cells, and Tregs and CD4 conventional T cells) [44, 45] and inflamed and non-inflamed gut [46] than there are between the lamina propria and epithelium [44–46]. Our study explores the effects of both the compartment (epithelium or lamina propria) and the presence of inflammation in a spectrum of different CD4 T-cell subsets and concludes that the compartment is the primary driver of the transcriptomic profile of a human small intestinal CD4 T cell.

Our data further indicates that the increase in migrating/infiltrating CD69^low^ CD4 T cells constitutes a major change in the local T-cell population during inflammation in CD. In recent years, the therapeutic armamentarium of IBD has been extended to the anti-integrin therapies vedolizumab (anti-integrin α4β7) and etrolizumab (anti-integrin β7). Sphingosine 1 phosphate (S1P), CCR9, and MAdCAM-1 targeted therapies are also under investigation in clinical trials for IBD [47]. Based on our data, it might be beneficial to implement lymphocyte trafficking interfering strategies early in the disease to prevent migration/infiltration of these cells. This might also prevent (pathogenic) infiltrating cells to become Trm cells over time.

The transcriptomic differences of CD4 T cells between the epithelium and lamina propria could be due to a preprogrammed destination of T cells or to local factors inducing a compartment-specific profile in the same cell of origin. A recent study in mice showed that T cells receive compartment-specific imprinting for the lamina propria or epithelial compartment in the mesenteric lymph nodes [45]. However, another study in mice showed that the transfer of lamina propria-derived T cells will lead to the relocation of these cells to both the lamina propria and epithelium [48]. Furthermore, overlap in the TCR repertoire for lamina propria and epithelium CD8 Trm cells has been observed [14], and several studies have shown that T cells adapt to the local microenvironment [30, 49]. It is likely that both lymph node-originated compartment-specific imprinting and the local environment contribute to the transcriptomic profile of T cells. Nevertheless, the extensive transcriptomic differences due to the compartment of residence emphasize the importance of considering the lamina propria and epithelium as separate compartments when deciphering the pathogenic mechanisms of chronic mucosal inflammation. This distinct compartmentalization of CD4 T cells also suggests that maintaining and restoring mucosal integrity is of key importance to prevent inappropriate immune responses.

The predicted key regulators of CD4 T-cell adaptation to the epithelium included TRIM27 involved in limiting Th1/Th17 differentiation [40, 41]. The set of predicted regulators, among others, could explain the less pronounced Th-cell and more pronounced innate/cytotoxic profile of intraepithelial compared to lamina propria CD4 T cells. Furthermore, the bulk RNA-sequencing data indicates that cell cycling/death in the epithelium is tightly controlled and that local CD4 T-cell expansion during inflammatory responses is primarily observed in the lamina propria. Uncontrolled cell death and extensive local expansion in the epithelial layer could potentially disrupt the single-cell layer of epithelial cells and consequently impact mucosal barrier integrity. Altogether, these data indicate a tight regulation of broad (and untargeted) effector function and cell cycling of intraepithelial CD4 T cells, potentially preventing destructive tissue responses while allowing targeted action via cytotoxicity. That many predicted key regulators involved in the adaptation of CD4 T cells to the epithelium are chromatin remodelers suggests that the adaptation to the microenvironment is imprinted in these cells. Future (single-cell) epigenomic/chromatin accessibility sequencing (e.g. ATAC-sequencing) could help to elucidate the specific chromatin alterations that occur, and thus the compartment-specific imprinting that ensues.

Active disease in patients with ileal CD does not result in transcriptional changes in CD4 T cells within the lamina propria. However, compared to the non-inflamed lamina propria of control subjects, cell activation and stress responses were observed. This strongly suggests that in established CD there are pre-existing changes in the transcriptome of CD4 T cells across the mucosa. Future research should include newly diagnosed patients or prediagnostic samples (with the latter being hard to collect) to assess how CD4 T cells at baseline or before diagnosis are altered. This could be pre-onset changes as shown by the presence of an IBD-like microbiome in healthy co-twins at risk of developing IBD [50], the influence of (previous) administered medication as shown in the blood of IBD patients [51], or the effect of previous flares. The fact that CD patients with an ileocecal resection often experience relapses at the anastomosis [52] suggests that the gut mucosa of CD patients exhibits (pre-existent) changes, although the occurrence of relapses could also be (partly) caused by non-immune cell-related factors.

Our data indicate that the inflamed epithelium undergoes extensive changes with an upregulation of protein translation and activation of CD4 T cells in patients with CD. The limited transcriptional differences of ileal CD4 Trm cells upon inflammation suggest that infiltrating CD4 T cells might have a pathogenic role. The finding that compositional and not transcriptional changes constitute the main change occurring in the immune compartment in patients with active IBD is supported by recent findings of Kong *et al.* [53]. Intraepithelial Tregs, however, did adapt on transcriptional level by gaining an effector Treg profile. This indicates that the local environment at the epithelial border changes significantly. Tregs are known to suppress pro-inflammatory and/or regulate anti-inflammatory functioning of CD8 T cells, TCRγδ T cells, and innate lymphoid cells [54, 55], which reside in high abundance at the epithelial border [2, 3]. Additionally, Tregs have direct tissue-specific functions including aiding tissue repair [56] and/or via interaction with epithelial stem cells [57] thereby preserving/stimulating mucosal barrier integrity. The significant changes observed in the inflamed epithelium suggest that promoting (effector) Treg differentiation and expansion in the gut mucosa as therapeutic strategy might be worthwhile. Indeed, a phase 1/2a clinical trial has shown that injection of antigen-specific Tregs in patients with CD resulted in a significant reduction of disease activity [58].

CD4CD8αα T cells are commonly referred to as cytolytic T cells derived from CD4 T cells that lost ThPOK and gained RUNX3 and T-bet expression and are primarily found in the (small intestinal) epithelium of mice [28, 48]. Murine small intestinal intraepithelial CD4CD8αα T cells aid in controlling local inflammatory responses via IL-10 and TGFβ [15, 29]. Human intestinal CD4CD8αα T-cell studies are limited, with one study showing a potential regulatory role via IL-10, CTLA4, GITR, CD25, and LAG3 in the colonic lamina propria [16]. Our data revealed no difference in the relative presence of ileal CD4CD8αα T cells between the epithelium and lamina propria, or between inflamed and non-inflamed mucosa. Furthermore, downregulation of *ZBTB7B* (encoding ThPOK) was not observed in CD4CD8αα T cells. Our study suggests that CD4CD8αα T cells in the human ileum are (very similar to) Trm cells, and do not express a clear cytotoxic or regulatory transcriptional profile.

Limitations of our study include assessment of inflammation during endoscopy without histological confirmation. Crohn’s disease is known for its heterogeneous nature with skip lesions resulting in alternation of inflamed and non-inflamed. Studies have shown that the correlation between endoscopic assessment and histological assessment of active disease is variable [59]. This problem can only be overcome if the same biopsy can be used for both immune cell mapping and assessment of histological disease activity. Employing spatial transcriptomics in which the whole immune compartment can be mapped as well as histological parameters to assess active disease to correlate findings would therefore be useful in future studies. Furthermore, active disease is assessed by the presence of neutrophils and not lymphocytes within the mucosa [60]. It is known that there is crosstalk between neutrophils and T cells [61]; however, the relationship between the presence of neutrophils and the phenotype as well as the transcriptome of T cells remains uncertain. Due to the small number of patients included we could not correlate disease state, use of medication, or other patient-specific characteristics to our findings. Inclusion of patients with on average mild and not severe endoscopic disease activity might have influenced our findings, but inclusion of patients with severe active disease might have prevented reliable study of intra-epithelial T cells due to destruction of the epithelial layer. Moreover, in peripheral blood it has been shown that different medications such as thiopurines and anti-TNF can have an opposite effect on the composition of the immune cell compartment [51]. Half of the included patients with Crohn’s disease did not use medication, but the other half used thiopurines, anti-TNF, 5-ASA, and/or steroids which could have made our results more heterogeneous. We observed few differences between the inflamed and non-inflamed ileum of patients with Crohn’s disease, but there were extensive differences between the inflamed ileum of patients with Crohn’s disease and the non-inflamed ileum of control subjects. This could indicate that the transcriptome of T cells residing in the intestinal mucosa in patients with Crohn’s disease has undergone changes independent of the presence of inflammation. However, it could also indicate that the distance of >2 cm for taking biopsies of inflamed and non-inflamed mucosa is insufficient and that there might be more differences if the biopsies would have been taken further apart. Also here, spatial information would be useful. Of note, the absence of transcriptomic differences does not mean the absence of relevant phenotypic differences. The use of *de novo* Crohn’s disease patients will be valuable to elucidate whether there is any imprinting of the T-cell transcriptome in patients with Crohn’s disease independent of inflammation.

In conclusion, our data reveal that there is a CD4 T-cell compartment-specific imprinting. Epithelial adaptation seems to result in an overall dampening of effector function and tight regulation of the lifespan. Furthermore, inflammation in patients with CD results in a relative increase of migrating/infiltrating CD69^low^ CD4 T cells, CD4 T-cell activation, and IFNγ responses as well as a strong effector Treg profile within the epithelium. These data indicate that the lamina propria and epithelium of the human ileum are differentially regulated in both control subjects and CD patients, with a pronounced regulatory role in the inflamed epithelium. Our findings suggest that we should consider both compartments in the therapeutic management of IBD.

## Supplementary Material

uxad095_suppl_Supplementary_Figures

uxad095_suppl_Supplementary_Table_S1

uxad095_suppl_Supplementary_Table_S2

uxad095_suppl_Supplementary_Table_S3

## Data Availability

The data underlying this article are available on https://github.com/lutterl/CD4-T-cells-ileum.

## Abbreviations:

CD: Crohn’s disease
FACS: fluorescence-activated cell sorting
FDRq: FDR statistical value
GSEA: gene set enrichment analysis
I: inflamed
IBD: inflammatory bowel diseases
IEL: intraepithelial T cell
LPL: lamina propria T cell
NES: normalized enrichment score
NI: non-inflamed
PI3K: phosphatidylinositol-3-kinase
SES-CD: simple endoscopic score for Crohn’s disease
TFs: transcription factors
Th: T helper
TRAF: TNF receptor-associated factor
Treg: regulatory T cell
Trm: Tissue resident memory
UMI: Unique molecular identifier.
