# Supplementary material for: Compartment-driven imprinting of intestinal CD4 T cells in inflammatory bowel disease and homeostasis
Source: Clin Exp Immunol. 2023 Aug 11;214(3):235–48. doi: 10.1093/cei/uxad095 (PMC10719222; doi:10.1093/cei/uxad095)
Supplement: uxad095_suppl_Supplementary_Figures [file uxad095_suppl_supplementary_figures.docx]

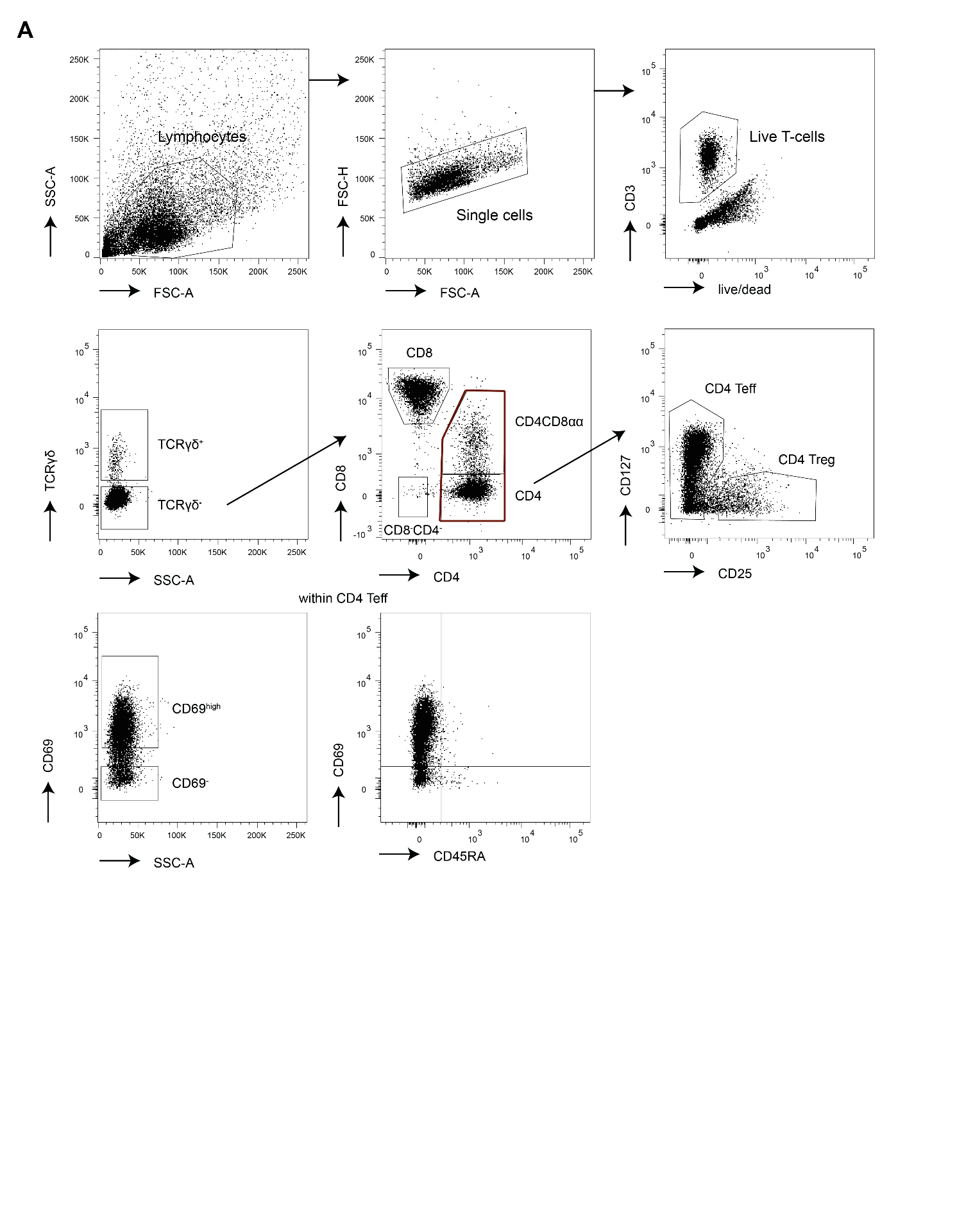


Supplementary figure 1. **Gating strategy.** (A) Gating strategy employed to determine the T cell subset composition, for sorting of CD4^+^CD69^high^, CD4^+^CD69^-^, CD4^+^ Treg, CD4CD8αα (CD8α^+^, vast majority is CD8αα) T cells for bulk RNA-sequencing and CD4^+^ T cells (red outline) for single-cell RNA-sequencing, and to determine the CD69/CD45RA composition. TCR, T cell receptor; Teff, T-effector cells; Treg, regulatory T cells.


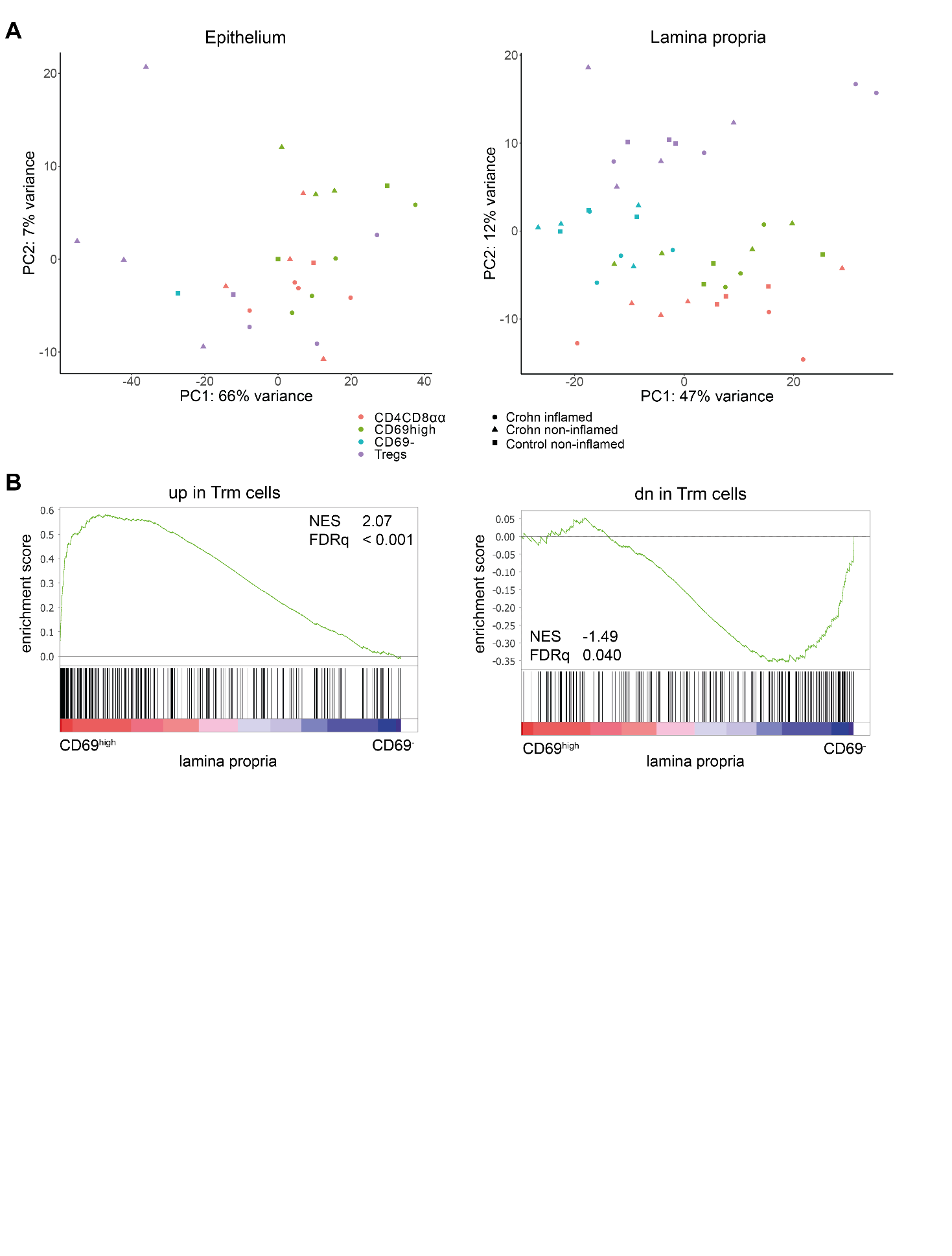


Supplementary figure 2. **Clustering of CD4 T cell subsets in the epithelium and lamina propria.** (A) Unsupervised principal component analysis of all sorted CD4 T cell subsets analyzed by bulk RNA-sequencing, split for epithelium (left) and lamina propria (right), colored on CD4 T cell subset (pink = CD4CD8αα T cells, green = CD4 CD69^high^ Trm cells, blue = CD4 CD69^-^ T cells, purple = Tregs) and status (circle = CD inflamed ileum, triangle = CD non-inflamed ileum, square = control non-inflamed ileum). (B) Gene set enrichment analysis of a Trm signature^4^ (CD69^+^ vs CD69^-^ T cells) with genes upregulated (left) and downregulated (right) in this signature in pairwise comparisons involving transcriptome data of CD69^high^ and CD69^-^ CD4 T cells derived from non-inflamed ileum of control subjects and non-inflamed and inflamed ileum of patients with CD, represented by the normalized enrichment score (NES) and FDR statistical value (FDRq, multiple hypothesis testing using sample permutation). CD inflamed/non-inflamed *n* = 4, control *n* = 3.
